# Generation of Flat Top Surface Plasmon Polariton Beams by Near Field Holography

**DOI:** 10.3390/nano9101377

**Published:** 2019-09-26

**Authors:** Peizhen Qiu, Chunyan Bai, Taiguo Lv, Dawei Zhang

**Affiliations:** 1 Department of Applied Physics, Huzhou University, Huzhou 313000, China; qiupeizhen@126.com; 2 Engineering Research Center of Optical Instrument and System, The Ministry of Education, Shanghai Key Laboratory of Modern Optical System, University of Shanghai for Science and Technology, Shanghai 200093, China; baichunyan1984@163.com (C.B.); lvtaiguo@lcu.edu.cn (T.L.); 3 School of Physics Science and Information Technology, Liaocheng University, Liaocheng 252000, China

**Keywords:** surface plasmonic polariton, flat top SPP beams, near field holography

## Abstract

Controlling the shape and trajectory of the surface plasmon polariton (SPP) beams is the key to all SPP-based applications. In this paper, a novel plasmonic device that can generate in-plane flat top SPP beams is designed by near field holography. The relationship between the transverse profile intensity of the generated flat top SPP beams and the structural parameters of the designed device is analyzed. The results of this paper can provide the possibility for further practical application utilizing flat top SPP beams.

## 1. Introduction

Surface plasmon polaritons (SPPs) are two-dimensional electromagnetic waves that propagate along the metal and dielectric interface. These surface waves originate from the strong interaction between free electron oscillations in the metal and external electromagnetic waves [1,2,3]. Owing to its strong enhancement of local field intensity and confinement of light energy to the sub-wavelength scale, SPPs have become a powerful tool for guiding and manipulating light below the diffraction limit [4]. Controlling the shape and trajectory of SPP beams is the key to all SPP-based applications [5]. Recently, researchers have designed a series of devices that can generated customized in-plane SPP beams, such as non-diffracting collimated SPP beams [6], Bessel-like SPP beams [7,8,9,10,11], Cosine-Gauss SPP beams [12,13,14], Mathieu and Weber or Airy SPP beams [15,16,17,18,19,20], SPP bottle beams (or SPP void arrays) [21,22,23,24].

Flat top beams, which have been widely studied in the free space, exhibit a sharp square transverse profile along the direction perpendicular to their propagating direction [25,26,27]. However, so far, flat top beams have not been widely explored in the field of surface plasmon photonics. In 2017, Manjavacas et al. deduced a rigorous theoretical description of flat top SPP beams, and theoretically confirmed the feasibility of the existence of flat top SPP beams at the interface between flat metal and dielectric [28]. It is observed that flat top SPP beams can be applied to achieve uniform excitation and coupling scenarios. However, that paper did not present what devices can be used to generate flat top SPP beams.

In order to solve this scientific problem, in this paper, we focus on what kind of device can be used to generate the flat top SPP beams. Near field holography technology is introduced to design the novel plasmonic device. As far as we know, this is the first time that a plasmonic device that can generate flat top SPP beams has been designed. The designing principle and the structure of the device are given. The effect of structure parameters of the designed device on the quality of the generated flat top SPP beams is investigated. The obtained results show that uniform excitation of flat top SPP beams can be realized by the proposed device.

## 2. Design Principle, Simulation Results and Discussion

In this section, the design principle to realize the proposed plasmonic device is firstly expounded. Secondly, the corresponding simulation results are given and discussed.

Recently, the concept of holography was introduced into the field of surface plasmonic photonics, which is referred to as near field holography (NFH) [29,30]. In the NFH method, SPP beams are used to participate in the holographic interference process. The two-dimensional binary holographic structure designed by NFH is used as a coupling structure, which can convert the free space beams to SPP beams. The near field holographic structure can be obtained by [31],

(1) $$t(x,y)=\frac{h_{o}}{2}\{1+sgn[\cos(\frac{2\pi }{\lambda_{spp}}x)-\cos[\pi q(x,y)]]\},$$

where *t(x,y)* represents the shape of the device on the *x-y* plane, *h*_o_ denotes the height of the designed structure in the *z* direction. sgn represents the symbolic function in Mathematic, *q(x,y)* contains the amplitude information of the target SPP beams, and *q(x,y)* = arcsin[*A*(*x*, *y*)]/π, where *A*(*x*,*y*) denotes the amplitude of the target SPP beams. *λ*_spp_ denotes the wavelength of the target SPP beams. The holographic structure lies in *(x,y)* plane. The *x*-axis denotes the SPP beam propagation direction and *y*-axis denotes the transverse direction. The two-dimensional holographic structure can be calculated by introducing the amplitude information of the target SPP beams into Equation (1). From Equation (1), one can see that the calculated holographic structure is periodically modulated with *λ*_spp_ in the *x*-axis and is determined by the amplitude of the target SPP beams in the *y*-axis.

Here, the NFH method is introduced to design a device that can generate flat top SPP beams. To examine the performance of the designed device, the finite difference time domain (FDTD) method, implemented by commercial software ‘FDTD Solutions’(produced by Lumerical Solutions Co. Ltd., Vancouver, BC, Canada), is used. The corresponding simulation results of the flat top SPP beams generated by the designed device are given. The effect of structure parameters of the designed device on the quality of the generated flat top SPP beams is studied.

**Figure 1:**
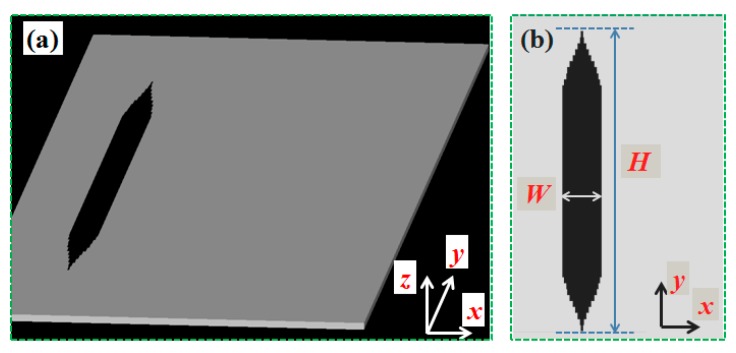
Schematic diagrams of the designed plasmonic device capable of generating flat top SPP beams, (**a**) three-dimensional configuration, with the height of *h*_o_ = 150 nm in *z* axis, (**b**) two-dimensional configuration. The black area indicates the cross-section shape of the groove that etched in the metallic silver film. *W* and *H* indicate the maximum length of the groove along *x* and *y* axis, respectively.

The definition of the three-dimensional coordinate axis is shown in Figure 1. Assuming at the position *x* = 0, the expression of a flat top profile (i.e., a square signal) can be described by [25,28],

(2) $$A(0,y)=e^{-y^{2}/w_{o}^{2}}\sum_{k=0}^{N}\frac{1}{k!}{\left(\frac{y}{w_{o}}\right)}^{2k},$$

where *w_o_* determines the width of a flat top profile, *N* determines the uniformity of a flat top profile. From the viewpoint of mathematics, larger values of *N* will lead to a much flatter profile. Especially when *N* = 0, the profile described by Equation (2) is a Gaussian profile. Now, substitute Equation (2) into Equation (3), and a plasmonic holographic structure (device) capable of generating flat top SPP beams is designed. Figure 1 shows the schematic diagrams of the device designed by combining Equations (1) and (2), when *N* = 22 and *w_o_* = 1.22*λ*_spp_. The other design parameters are as follows. The wavelength of incident light is *λ*_o_ = 1.55 μm. The thickness of the silver film *h*_o_ = 150 nm. The dielectric permittivity of the air and the silver are respectively *ε*_d_ = 1 and *ε*_m_ = −129.17 + *i*3.2841 at 1.55 μm [32]. Thus, the corresponding wavelength of the generated SPP beams is *λ*_spp_ = 1.544 μm. Figure 1a shows the three-dimensional configuration of the designed plasmonic device. Figure 1b shows the two-dimensional configuration of the designed plasmonic device on the *x*–*y* plane. The incident light normally illuminates the device from the bottom of the device. The incident light is linearly polarized plane wave and its polarization direction is along the *x*-axis. In Figure 1b, the black area indicates the cross-sectional shape of the groove that is etched in the metallic silver film, showing a rectangular shape in the middle and a tip-shaped shape at both ends. The depth of the groove is the same as *h*_o_. The shape of the groove on the x-y plane remains unchanged at any position on the *z*-axis (in the range of *h*_o_). In Figure 1b, *W* denotes the width of the groove in the *x* direction and *W* = 0.772 μm. *H* denotes the length of the groove in the *y* direction and *H* = 20.2 μm, wherein the length of the middle rectangle is approximately 13 μm. The value of *W* is half of the *λ*_spp_. *H* is determined by *w_o_* shown in Equation (2). In order to reduce the computational complexity of simulation, only a single groove is used as the device structure and shown in Figure 1a. In fact, from Equations (1) and (2), periodic groove arrays with period of *λ*_spp_ along the *x*-axis can be obtained. When the structure shown in Figure 1 is normally illuminated by the incident lights, the first diffraction order of the holographic plaomonic structure can be coupled into flat top SPP beams.

For more detailed working principles of the NFH method, on can see [30,31]. Here follows a brief introduction to how to couple free space beams to SPP beams using devices designed by Equation (1). In NFH, the reference beam is the free space beam, and the reconstructed beam is the target plasmonic beam (here is the flat top SPP beam). As we know, the wave vector of SPP beams *k*_spp_ is larger than that of the free space beams *k*_o_. Thus, there exists a missing momentum between these two beams. In order to excite SPP beams, a common method to contribute the missing momentum is to use a grating structure with period *λ*_spp_. A single groove with a special shape that is etched in a metal film can be regarded as a special grating. Thus, the principle of grating diffraction can be used to explain how the holographic structure can convert free space beams into SPP beams. When the structure is normally illuminated by the incident free space light, the first diffraction order of the structure is coupled into SPP beams.

**Figure 2:**
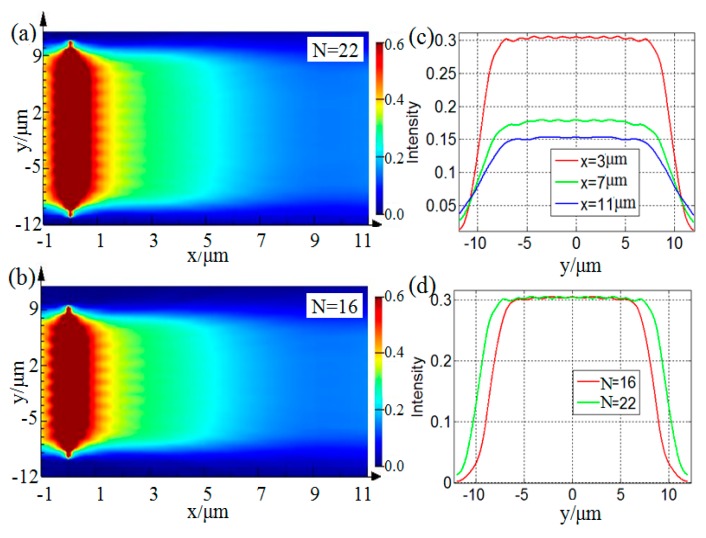
The simulated intensity of the flat top SPP beams with *w_o_* = 1.22λ_spp_, (**a**) *N* = 22, (**b**) *N* = 16. (**c**) The transverse profile intensities extracted from (**a**) at *x* = 3, 7, 11 μm. (**d**) The transverse profile intensity distributions at *x* = 3 μm extracted from (**a**) and (**b**).

Two devices capable of generating flat top SPP beams are designed by combining Equations (1) and (2). The design parameters are, *w_o_* = 1.22*λ*_spp_, *N* = 22 or *N* = 16. From Equation (2), one can see that the values of *N* and *w_o_* will affect the structure of the designed device, and further affect the quality of the flat top SPP beams generated by the proposed device [28]. Figure 2a,b shows the simulated results of the intensity distributions of the generated flat top SPP beams, when *N* = 22 and *N* = 16, respectively. In order to quantitatively study the propagation characteristics of the flat top SPP beams, the transverse profile intensities of the generated flat top SPP beams at propagation distances of *x* = 3, 7, 11 μm are extracted from Figure 2a and plotted in Figure 2c. It can be seen that, as the propagation distance increases, the transverse profile width of flat top SPP beams gradually increases, and the transverse profile intensity gradually decreases, which are caused by the divergence of light during propagation. In addition, the transverse profile intensity distributions of the flat top SPP beams shown in Figure 2a,b at a propagation distance of *x* = 3 μm are extracted and plotted in Figure 2d. One can see that, as *N* increases, the transverse profile width of the flat top SPP beams increases, but the edges on both sides of the transverse profile intensity distribution curve become steeper.

To further quantitatively analyze the effect of different *N* values on the transverse intensity distributions of the generated flat top SPP beams, the concept of kurtosis is introduced. Kurtosis can be used to quantify the uniformity of the flat top SPP beams, which can be defined as [28],

(3) $$K(x)=\frac{\langle y^{4}\rangle }{{\langle y^{2}\rangle }^{2}},with\langle y^{n}\rangle =\frac{\int_{-\infty }^{\infty }y^{n}{\left|f(x,y)\right|}^{2}dy}{\int_{-\infty }^{\infty }{\left|f(x,y)\right|}^{2}dy},$$

where |*f*(*x*, *y*)|^2^ denotes the intensity distribution of the generated flat top SPP beams. Assuming that the distribution curve is a perfect rectangular distribution, the corresponding *K*(*x*) = 1.8 can be obtained by Equation (3), which is utilized as a reference kurtosis. The closer the value of kurtosis is to 1.8, the larger uniformity of the transverse profile of the generated flat top SPP beams is.

**Figure 3:**
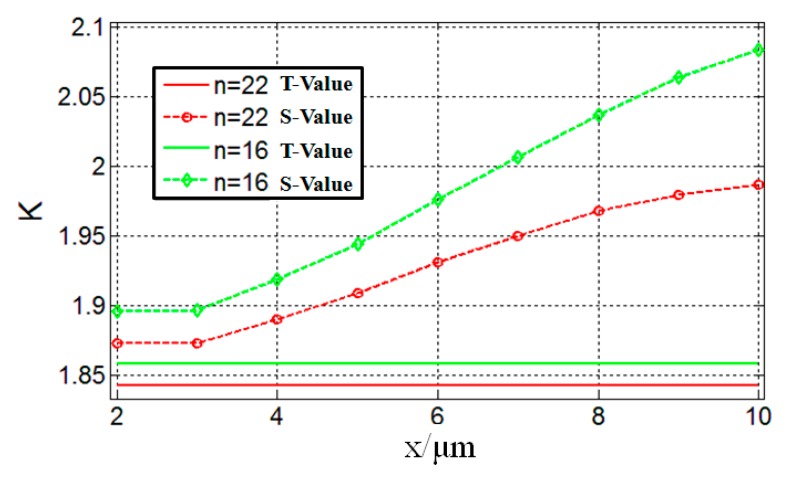
Evolution of the kurtosis for two different flat top SPP beams as a function of the propagation distances. *w_o_* = 1.22*λ*_spp_, *N* = 22 (red curves) and *N* = 16 (green curves). The solid curves represent the theoretical values (T-Values) obtained by Equations (2) and (3), for *N* = 22, *K* = 1.8422 (red solid curve), for *N* = 16, *K* = 1.8579 (green solid curve). The dotted curves represent the simulation values (S-Values) obtained by FDTD software.

The evolutions of the kurtosis for two different flat top SPP beams with different propagation distances have been studied. In Figure 3, the red curves correspond to the case of *N* = 22, *w_o_* = 1.22*λ*_spp_. Among which, the red solid curve represents the reference kurtosis, *K_N_*_=22_ = 1.8422, which is calculated by Equations (2) and (3). The red dotted curve represents the simulation values obtained by FDTD software. The calculation procedures are as follows: Firstly, the transverse profile intensity distributions *f*(*x*,*y*) of the generated flat top SPP beams at different propagation distances are extracted from Figure 2a. Secondly, the corresponding kurtosis at different propagation distances is calculated by substituting *f*(*x*,*y*) into Equation (3). Then, the evolution curve of kurtosis as a function of propagation distances can be plotted. The green curves shown in Figure 3 corresponds to the case of *N* = 16, *w_o_* = 1.22*λ*_spp_. Using a similar calculation approach, the green solid curve represents the reference kurtosis, *K_N_*_=16_ = 1.8579. The green dotted curve represents the simulation values for the case *N* = 16. As can be seen from Figure 3, larger values of *N* results in lower values *K*, which are closer to the reference kurtosis *K* = 1.8, reflecting the larger uniformity of the transverse profile intensity distributions of the generated flat top SPP beams. In addition, regardless of whether *N* = 22 or *N* = 16, the kurtosis gradually increases as the propagation distance increases. This is due to the divergence of light in the propagation process, resulting in the broadening of the transverse profile of the flat top SPP beams.

**Figure 4:**
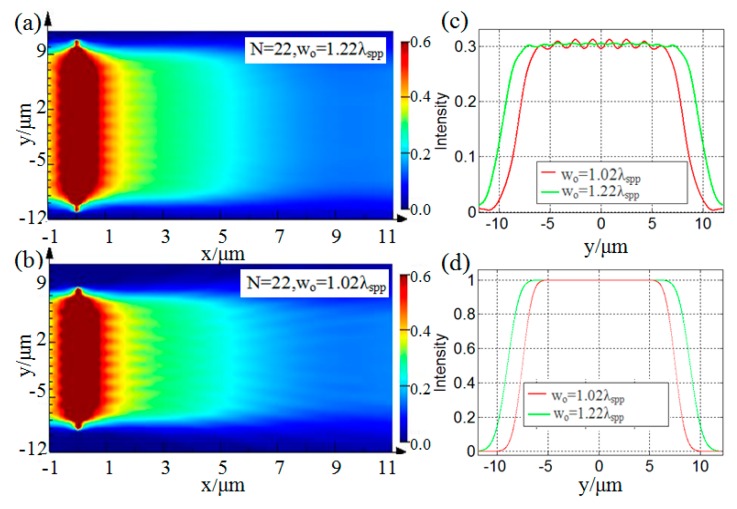
The intensity distributions of the generated flat top SPP beams under *N* = 22, (**a**) *w_o_* = 1.22λ_spp_, (**b**) *w_o_* = 1.02*λ*_spp_. (**c**) A comparative diagram of the transverse profile intensity distributions extracted at *x* = 3 μm from (**a**) and (**b**). (**d**) A comparative diagram of the transverse profile intensity distributions calculated by Equation (1).

Figure 4 shows the simulated intensity distributions of the generated flat top SPP beams when *N* = 22, (a) *w_o_* = 1.22*λ*_spp_ and (b) *w_o_* = 1.02*λ*_spp_. By comparing Figure 4a,b, one can see that, with an increase of the value *w_o_*, the transverse size of the corresponding designed device increases, and the transverse width of the corresponding generated flat top SPP beams also increases. To further analyze the characteristics of the generated flat top SPP beams under different values of *w_o_*, the transverse profile intensity distributions at *x* = 3 μm shown in Figure 4a,b are extracted and plotted in Figure 4c. Correspondingly, Figure 4d shows a comparative diagram of the transverse profile intensity distributions directly calculated by Equation (1). It can be seen that Figure 4c,d shows good consistency. With the increase of *w_o_*, the beam width increases. Thus, it can be concluded from the above results that the changes in the values of *N* and *w_o_* will affect the structure shape and size of the designed device, which in turn affect the quality of the flat top SPP beams generated by the designed device. The larger the value of *N*, the flatter the transverse profile of the corresponding generated flat top SPP beams. The larger the value of *w_o_*, the wider the transverse profile of the flat top SPP beams. Therefore, the width and uniformity of the transverse profile of the generated flat top SPP beams can be controlled by changing the values of *N* and *w_o_*. These conclusions are in good agreement with the previous theoretical studies in [28], which indirectly proves the correctness of the functionality of the device designed by the NFH method in this paper. In addition, the relevant simulation results demonstrated that the feasibility of the designed device in generating flat top SPP beams. Similar to [28], we mainly use kurtosis and width as criteria to evaluate the quality of the generated flat top SPP beams. As indicated in [28], there is a trade-off between uniformity and divergence which can be controlled by the value of *N* and *w_o_*. In general, *N* controls the uniformity, and *w_o_* controls the width. The increase of either of *N* and *w_o_* will result in a larger divergence. Therefore, in practical applications, we should appropriately choose and optimize the values of *N* and *w_o_*, according to the actual needs.

## 3. Conclusions

In this paper, to our best knowledge, a novel plasmonic device that can generate flat top SPP beams is designed for the first time. This is also the first time near field holography technology has been used to design a device that can generate flat top SPP beams. The relationship between the transverse profile of the generated flat top SPP beam and the structural parameters of the designed device is discussed. Two parameters—width and kurtosis—are utilized to analyze the propagation characteristics of the generated flat top SPP beams. By changing the values of *N* and *w_o_*, the width and uniformity of the transverse profile of the generated flat top SPP beams can be controlled. The corresponding numerical simulation results prove the feasibility of generating flat top SPP beams based on the device designed by NFH method.

This paper focuses on what kind of device can be used to generate the flat top SPP beams. This is the main difference between this work and [28]. The results of this paper verify the correctness of theoretical predictions in [28] for the existence of propagating flat top SPP beams at the dielectric-metal interface from the perspective of device design. Similar to the definition of free-space flat top beams, flat top SPP beams also exhibit a constant transverse profile perpendicular to their propagation direction. Thus, the flat top SPP beams can be applied to achieve uniform excitation and coupling scenarios, which cannot be realized by traditional Gauss SPP beams.

In end, the results of this paper can provide the possibility for practical application of using the flat top SPP beams. In addition, the flat top SPP beams generated by the proposed device in this paper enrich the types of SPP beam and can further improve the ability of researchers to utilize SPP to control and manipulate light below the diffraction limit.
